# The Role of the Immune System in the Course of Hashimoto’s Thyroiditis: The Current State of Knowledge

**DOI:** 10.3390/ijms25136883

**Published:** 2024-06-23

**Authors:** Karolina Wrońska, Maciej Hałasa, Małgorzata Szczuko

**Affiliations:** 1 Department of Human Nutrition and Metabolomics, Pomeranian Medical University in Szczecin, 71-460 Szczecin, Poland; 74985@student.pum.edu.pl; 2 Department of General Pathology, Pomeranian Medical University in Szczecin, 71-204 Szczecin, Poland; maciupam@op.pl

**Keywords:** Hashimoto’s disease, B lymphocytes, T lymphocytes, natural killer, T helper cells, regulatory T cells, apoptosis, pyroptosis

## Abstract

The process of thyroid autoimmunization develops against the background of genetic predispositions associated with class II human leukocyte antigens (HLA-DR), as well as cytotoxic T-lymphocyte-associated protein 4 (CTLA-4), protein tyrosine phosphatase non-receptor type 22 (PTPN22), and forkhead transcription box protein P3 (FOXP3). Environmental factors, such as vitamin D deficiency, Zn, Se, and Mg, as well as infections, chronic stress, pregnancy, smoking, alcohol, medications, intestinal dysbiosis, and malnutrition, also play an important role. The first stage of autoimmunization involves the accumulation of macrophages and dendritic cells, as well as plasma cells. In the second stage, the mutual interactions of individual cells in the immune system lead to a decrease in the level of CD8+ in favor of CD4+, which intensifies the synthesis of T lymphocyte derivatives, especially Th1, Th17, Tfh, and Tc, reducing the level of Treg. Consequently, the number of the anti-inflammatory cytokines IL10 and IL2 decreases, and the synthesis of the pro-inflammatory cytokines IL-2, Il-12, Il-17, IL-21, IL-22, IFN-γ, and TNF-α increases. The latter two especially trigger the pyroptosis process involving the inflammasome. Activation of the inflammasome by IL-β and IL-18 produced by macrophages is one of the mechanisms of pyroptosis in the course of Hashimoto’s thyroiditis, involving Gram-negative bacteria and NLRC4. In the next step, the apoptosis of thyroid cells is initiated by the intensification of perforin, granzyme, and proteoglycan synthesis by Tc and NK cells. The current findings raise many possibilities regarding interventions related to the inhibition of pro-inflammatory cytokines and the stimulation of anti-inflammatory cytokines produced by both T and B lymphocytes. Furthermore, since there is currently no effective method for treating thyroid autoimmunity, a summary of the review may provide answers regarding the treatment of not only Hashimoto’s thyroiditis, but also other autoimmune diseases associated with autoimmunity.

## 1. Introduction

Hashimoto’s thyroiditis (HT) is one of the most common endocrine disorders worldwide, affecting 1–2% of the population [1]. It should be emphasized that it occurs in patients of different ages, including children, and it is 10–20 times more common in women than in men [2]. It is an autoimmune disease with a complex pathogenesis that is not fully understood, which is associated with environmental and genetic factors [3]. Chronic lymphocytic thyroiditis is an example of organ-specific autoimmunization. CD4+ and CD8+ T cells, CD19+ B cells, macrophages, and plasma cells attack the thyroid gland, resulting in the destruction of thyrocytes and the thyroid epithelial structure. T helper cells (Th), especially Th1 and Th17, also play a significant role in the development of the disease [4]. At the same time, the production of thyroid hormones is impaired. Autoreactive T cells, but also B lymphocytes, may play crucial role in the development of the disease. These cells are the main source of autoantibodies to thyroid peroxidase and thyroglobulin [1]. The presence of antithyroid peroxidase antibodies (anti-TPO) is a clinical marker that confirms the presence of the disease. Thyroid ultrasonography (USG) may be helpful in the diagnosis, especially in patients with no autoantibodies detected in the blood [5]. Thyroid imaging changes suggestive of Hashimoto’s thyroiditis include decreased echogenicity, heterogeneity, excessive vascularity, and the presence of small cysts [5]. Characteristic manifestations of HT include the fibrosis and atrophy of the thyroid parenchyma. The review of current scientific research presented in this article aims to increase awareness of the role of the immune system in the pathogenesis of Hashimoto’s thyroiditis and to emphasize the importance of monitoring the levels of individual lymphocyte subpopulations in the diagnosis, treatment, and assessment of disease progression in patients.

## 2. Epidemiology, Etiology, and Pathogenesis of Hashimoto’s Thyroiditis

An increase in the incidence of Hashimoto’s thyroiditis is currently being observed; therefore, knowledge of the etiology and pathogenesis of the disease is crucial to the diagnostic and therapeutic process [6]. Women suffer from Hashimoto’s disease 7–10 times more often than men. The highest incidence is observed between 30 and 60 years of age. However, it is noteworthy that HT can occur in patients at any age, even affecting children. The disease can manifest as clinically overt, occurring in approximately 0.1–2% of the population. The subclinical form is more common, affecting 10–15% of the population [2]. Ethnic origin influences the risk of developing the disease [5]. It has been shown that the disease is diagnosed significantly more often in individuals of Caucasian ethnicity. Black and Asian people have a lower incidence of the disease. People living in the Pacific Islands have a low incidence of chronic lymphocytic thyroiditis [5]. Many factors have been suggested that lead to an imbalance in immune tolerance mechanisms, including an excess or a deficiency of nutrients, exposure to heavy metals, toxins, and endocrine disruptors [2]. It has also been shown that identical twins and women born in the summer have a higher risk of developing the disease [7]. Thyroid autoimmunity is influenced by past infections, hepatitis C virus, chronic stress, pregnancy, smoking, alcohol consumption, medications used, e.g., interferon-alpha, amiodarone, and lithium, and the gut microbiota [8].

### 2.1. Genetic Factors

Genetic and environmental factors play the most important role in pathogenesis of the disease, leading to the provocation of the immune system and the increased production in antibodies that destroy the thyroid gland. Many immune response genes have been identified as playing a significant role in the development of HT. Polymorphisms of class II human leukocyte antigens (HLA-DR) genes, as well as cytotoxic T-lymphocyte-associated protein 4 (CTLA-4), cluster of differentiation 40 (CD40), protein tyrosine phosphatase non-receptor type 22 (PTPN22), forkhead transcription box protein P3 (FOXP3), and CD25, disrupt the mechanisms of immune tolerance [6,9]. It has been shown that alterations in these genes lead to T cell activation and influence antigen presentation. The interaction of T cells with antigen-presenting cells (APCs) may also be abnormal due to polymorphisms in the immunoregulatory genes [10]. In the pathogenesis of Hashimoto’s thyroiditis, abnormalities in regulatory T cells (Treg) and T follicular helper cells (Tfh) have also been implicated in the initiation and progression of chronic lymphocytic thyroiditis. Accumulating lymphocytes have a cytotoxic effect on thyrocytes, disrupting their metabolic progression activity. DNA fragments that remain after cell death also influence the initiation of the disease [6,10].

### 2.2. Environmental Factors: Nutrition

Chronic iodine excess increases the immunogenicity of thyroglobulin, leading to the stimulation of apoptosis-related mechanisms [9]. It also influences the increased production of Tc cells that attack the thyroid gland and lead to the cessation of regulatory T cell development, resulting in gland damage [10]. Iron deficiency has also been shown to increase the immunogenicity of thyroid peroxidase and thyroglobulin [10]. Selenium deficiency in the body increases T cell activity, disrupts the balance between Th1 and Th2, leads to excessive cytokine production such as tumor necrosis factor-alpha (TNF-α), and reduces the number of regulatory T cells. Selenium supplementation has been shown to significantly reduce the levels of antibodies in thyroid peroxidase. Vitamin D supplementation is also beneficial [8]. Too little vitamin D results in the increased differentiation of B lymphocytes into plasma cells that produce antibodies [10]. Other risk factors for Hashimoto’s thyroiditis include magnesium and zinc deficiency.

### 2.3. Gut Dysbiosis

**Figure 1:**
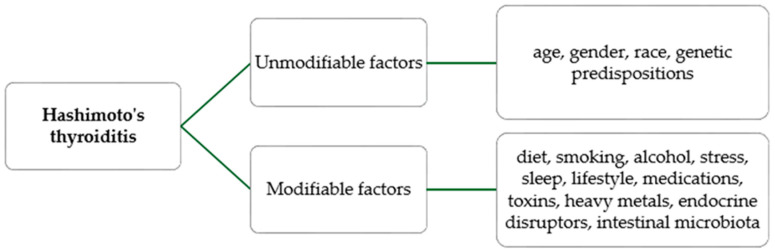
Risk factors for Hashimoto’s thyroiditis.

Dysbiosis increases the risk of developing inflammatory diseases resulting from autoimmunity. Clinical studies in patients have shown a decrease in beneficial bacteria such as *Bifidobacterium* and *Lactobacillus* and an increase in *Bacteroides fragilis* [11]. Although Hashimoto’s thyroiditis affects an increasing number of people and the number of studies on the subject is increasing, further research is necessary to expand our knowledge and determine the relevant preventive and therapeutic goals [6]. The factors influencing the occurrence of Hashimoto’s thyroiditis are presented below (Figure 1).

## 3. Diagnosis

Hashimoto’s thyroiditis is diagnosed based on of the clinical symptoms of thyroid hypofunction and biochemical evidence of the presence of antithyroid peroxidase antibodies and/or antithyroglobulin antibodies (anti-TG) [12]. Antithyroid peroxidase antibodies are present in approximately 95% of patients, and antithyroglobulin antibodies are present in 60–80% of patients [5]. In a study conducted in India by Thomas et al., which involved 144 patients diagnosed with HT, anti-TPO antibodies were detected in 93% and anti-TG in 92% of cases [13]. Monitoring antibody levels over the course of the disease may be helpful in determining the risk of progression to overt hypothyroidism [5]; it also plays a role in decisions related to disease management [6]. In women diagnosed with Hashimoto’s thyroiditis who are trying to get pregnant, the level of antithyroid peroxidase antibodies provides important information about the risk of hypothyroidism during pregnancy, miscarriage, or the failure of in vitro fertilization procedures [5]. When diagnosing the disease, it is important to note that 5–10% of patients do not have detectable levels of these antibodies [5]. Over the course of lymphocytic thyroiditis, the presence of antibodies against sodium iodide symporter (NIS) and pendrin may also be observed, although their clinical significance has not been confirmed [6]. Although a fine-needle aspiration biopsy (FNA) is regarded a key test for confirming or excluding the presence of HT, it is not always feasible in actual clinical practice. It may be crucial for differential diagnosis, especially when no antibodies are detected in the blood [12]. Ultrasonography is another diagnostic method used in patients. It allows not only for the detection of characteristic nodules, but also for the assessment of the thyroid tissue’s echogenicity and vascularity [12]. The assessment of the thyroid’s appearance may be useful in the differential diagnosis, especially in patients with no detectable antibodies in their blood. In adults, among the characteristic features of HT that are visible on USG, there is glandular enlargement, the presence of fibrous septa, micro-nodularity, excessive vascularity, and decreased echogenicity. As the disease progresses, parenchymal atrophy of the thyroid gland is observed [12]. In addition to the aforementioned changes, patients also exhibit lymph node enlargement in the perithyroidal area. A study conducted by Kosiak et al. showed that the presence of lymph nodes adjacent to the lower parts of the thyroid lobes is a characteristic feature of the disease even in children, with a sensitivity of 98% and specificity of 100% [12]. Over the course of HT, lymphocytic infiltration and scarce colloid or its absence are observed [13]. The presence of thyroid cell infiltration is confirmed at different stages of progression. Plasma cells, which are essential for the diagnosis of the early stage of the disease when lymphocytic infiltration is minimal, are also detected [13].

## 4. Clinical Presentation and Treatment

Symptoms in patients with Hashimoto’s thyroiditis occur due to the high level of antibodies attacking thyroid antigens and the development of hypothyroidism. In euthyroidism, when TSH and thyroid hormone levels are normal, HT might have a non-specific course. Patients may notice non-severe symptoms such as mood swings, impaired concentration, dry skin, hair loss, fatigue, and weight gain [2]. These symptoms might be unrecognized by patients for a prolonged time. As the disease progresses and evolves into hypothyroidism, symptoms worsen and patients present with both local symptoms associated with thyroid enlargement and systemic symptoms [14]. Characteristic symptoms include fatigue, weight gain, constipation, difficulty concentrating, and depression [15]. Cold intolerance, dry skin, hair loss, mood swings, stress, and anxiety are other manifestations of thyroid hormone deficiency [14]. Over the course of the disease, bradycardia, decreased cardiac contractility, hyperlipidemia, anemia, menstrual irregularities, or infertility may also occur. Symptoms may persist despite thyroid hormone therapy in approximately 5–10% of patients [15]. In addition to systemic symptoms, patients may experience neck and throat pain and discomfort, voice changes, dyspnea, dysphagia, or sleep apnea. Laboratory abnormalities may be observed. Increases in creatine kinase, prolactin, total cholesterol, triglycerides, and LDL cholesterol have been reported [11]. Although immune mechanisms are important in the pathogenesis of the disease, the existing therapy is based solely on the patient’s thyroid status and function [16]. Patients with thyroiditis and transient hyperthyroidism, which usually leads to permanent hypothyroidism, are treated supportively [5]. In the case of overt hypothyroidism, levothyroxine therapy is required. The dose of levothyroxine depends on the patient’s hormone levels, age, and body weight, and the time of year [16]; it typically ranges from 1.6 to 1.8 micrograms per kilogram of body weight [8]. Regular use of thyroid hormone has been shown to decrease Tfh cell activity and increase Treg cell numbers [16]. In pregnant women, liquid levothyroxine treatment appears to be more beneficial [7]. Once therapy is initiated, it is essential to continuously monitor the treatment progress and patient status, and to adjust the dosage accordingly. Levothyroxine treatment in pregnant women diagnosed with Hashimoto’s thyroiditis has a beneficial effect on pregnancy outcomes and infant development. It reduces the risk of miscarriage, pregnancy-induced hypertension, gestational diabetes, pre-eclampsia, and delivery complications. However, combined thyroxine (T4) + triiodothyronine (T3) therapy is not recommended during pregnancy [5]. Another promising treatment involves the use of a specific histone deacetylase 6 inhibitor in patients, which results in reduced Th17 cell differentiation and less tissue damage to the thyroid [7]. In addition to pharmacological treatment, important elements of disease management include the following: a proper high fiber diet and adequate amount of nutrients, supplementation, and sleep [7]. Surgical intervention may also be considered for patients, especially in cases of severe pain, compression, or the presence of malignant thyroid nodules [5].

## 5. Health Risks and Long-Term Effects

Patients with Hashimoto’s thyroiditis may experience thyroid damage and hypothyroidism, which manifests with elevated levels of thyroid-stimulating hormone (TSH) and decreased levels of free thyroxine (FT4) and/or free triiodothyronine (FT3) in the blood [1]. The most severe form of advanced hypothyroidism is Hashimoto’s encephalopathy, a rare condition that can lead to coma [5]. Patients with subclinical hypothyroidism are at increased risk of cardiovascular disease. Properly managed thyroid hormone replacement therapy significantly improves patients’ health status and reduces the risk of heart attack, stroke, arrhythmia, heart failure, or cardiovascular-related death [11]. It should be emphasized that patients with HT are more likely to develop both cancerous and non-cancerous changes [17]. Furthermore, a significant increase in the risk of cardiovascular events and cardiovascular-related mortality has been demonstrated in patients with a TSH level of 10 mIU/mL or higher [5]. The presence of an autoimmune disease in a patient increases the risk of subsequent diseases resulting from auto-aggression. This is due to abnormal humoral and cellular responses to self-antigens [18]. Immune dysregulation and cross-reactions with different antibodies occur throughout the body [19].

### Other Autoimmune Comorbidities

The presence of common genes responsible for the development of autoimmune diseases is also a significant factor leading to the development of multiple diseases in the same patient [19]. One third of patients are also diagnosed with other non-thyroid diseases resulting from abnormal immune system function [20]. Hashimoto’s thyroiditis coexists with other diseases in the autoimmune polyendocrine syndromes (APS). This is a group of diseases resulting from autoaggression that causes the dysfunction of at least two endocrine glands. APS-2 and APS-3 are the ones most commonly diagnosed in patients. In APS-2, Hashimoto’s thyroiditis coexists with Addison’s disease, type I diabetes, hypogonadism, hypoparathyroidism, or pituitary insufficiency. In APS-3, chronic lymphocytic thyroiditis may occur with rheumatoid arthritis, lupus, Sjögren’s syndrome, or type I diabetes [21].

In recent years, an association between celiac disease and autoimmune thyroid diseases, including HT, has also been observed. The main link between the diseases is the presence of common genes in patients, such as HLA-B8, HLA-D3, HLA-DQ2, HLA-DQ8, CTLA-4, interleukin 18 (IL), and interferon gamma (IFN-y). Notably, most of these genes are components of the major histocompatibility complex (MHC), which stimulates the body’s immune response [22]. Kayar and Dertli observed that Hashimoto’s thyroiditis occurred in 17% of 230 individuals with celiac disease [23]. In cases of genetic predisposition to celiac disease and chronic lymphocytic thyroiditis, exposure of the immature immune system to gliadin leads to changes in the immune response, increasing the risk of these diseases [23]. It is essential that every patient with Hashimoto’s thyroiditis be screened for celiac disease in order to select the appropriate therapy and prevent serious health consequences. Another condition that coexists with HT is premature ovarian failure. During the disease, there is an infiltration of T cells, B cells, natural killer (NK) cells, and plasma cells [24]. Premature ovarian failure also occurs in APS-3, along with conditions such as Hashimoto’s thyroiditis. Studies have shown that autoimmune thyroid disease, most commonly lymphocytic thyroiditis, occurs in approximately 30% of women at the time of diagnosis and in the early stages of the disease. Patients exhibit both clinical symptoms of HT and the presence of anti-TPO and anti-TG antibodies. According to the guidelines of the European Society of Human Reproduction and Embryology, any patient with premature ovarian failure should be screened for anti-TPO antibodies. If antithyroid antibodies are detected in the blood, TSH levels and anti-TPO antibodies should be monitored annually [24]. Addison’s disease is another autoimmune disease that coexists with Hashimoto’s thyroiditis [19]. Morawiec-Szymonik et al. showed that almost half of the individuals with newly diagnosed pernicious anemia also had antithyroid antibodies, leading to the development of autoimmune thyroid disease. Antithyroid peroxidase antibodies were detected in 36.3% of the participants, and antithyroglobulin antibodies in 25%. Moreover, 20.2% of participants had both anti-TPO and anti-TG antibodies. It is essential to measure the concentration of antithyroid antibodies in the blood of every person with pernicious anemia in order to select an appropriate therapy and improve patients’ health status [19].

Patients with autoimmune diseases that coexist with Hashimoto’s thyroiditis should be diagnosed even if they have no symptoms [25]. The proper clinical management of patients with autoimmune diseases, risk assessments for other diseases, and comprehensive diagnostics are essential for detecting existing diseases as early as possible and optimizing treatment approaches [26].

## 6. Mechanism of Thyroid Cell Destruction

The disease’s pathogenesis continues to be studied, and groundbreaking discoveries are being made. However, many factors related to the immune system that lead to the onset and progression of the disease remain unexplained [27]. Certainly, a key factor in the initiation of the disease is the loss of tolerance, which can be caused by the breakdown of central tolerance or by disturbances in the maintenance of peripheral tolerance [28]. In the early stages of the disease, there is an accumulation of antigen-presenting cells expressing MHC class II in the thyroid. These are mainly dendritic cells and macrophages. As a result of the presentation of organ-specific autoantigens, naive CD4+ T-cells undergo activation and clonal expansion [28]. Subsequently, B cells and T cells that are capable of recognizing autoantigens are generated, leading to the production of antibodies and a progressive, irreversible process of thyroid destruction. Patients exhibit abnormalities in both cellular responses involving cytotoxic T cells and humoral responses related to antibody production. Three main mechanisms leading to thyroid destruction have been identified, as follows: cytotoxic T cells, death receptors, and antibodies [1]. The importance of decreased regulatory T cell activity and increased helper T cell activity is also emphasized [27]. A characteristic manifestation of the disease is an inflammatory infiltration within the thyroid gland, where the presence of CD4+ and CD8+ T cells, CD19+ B cells, and macrophages and plasma cells has been demonstrated [27]. The attack of these cells results in damage to the thyroid parenchyma, inflammation, fibrosis, reduced hormone production, and glandular hypofunction.

When analyzing the course of Hashimoto’s thyroiditis, it is also necessary to assess the role of humoral elements that regulate inflammation, which determine the course of the immune response [6]. Cytokines lead to the destruction of thyrocytes via many mechanisms at various stages of disease development. They affect the cells infiltrating the thyroid gland and modulate the maturation and activity of thyrocytes, which begin to produce factors that intensify inflammation, leading to the progression of the disease and the deterioration of the patient’s health and wellbeing. Particular attention should be paid to substances that are present in the lymphocytic infiltrate because they directly contribute to the destruction of the thyroid gland [11]. The pathogenesis of Hashimoto’s thyroiditis involves the involvement of cytokines produced by cells of the immune system, such as Th1, Th2, Th3, and Th17, which influence both cellular and humoral immunity [6]. It is worth mentioning the different effects of cytokines because, in specific situations, they can stimulate or inhibit the immune system. The studies assessed in this review have proven that IL-2 and TNF-α have variable effects, contributing to both the progression and decrease in the severity of the disease [27].

### 6.1. T Lymphocytes

T lymphocytes play a critical role in the pathogenesis of chronic lymphocytic thyroiditis, and their involvement is increasingly well understood. They are part of the specific immune system and participate in cell-mediated responses. Their role is to maintain order, balance, and protection against disease. T lymphocytes are divided into groups, the most important being CD4+ and CD8+ [29]. T-cells are able to recognize antigens presented by antigen-presenting cells through a receptor on their surface (TCR). Subsequently, naive T cells are stimulated to undergo clonal expansion and differentiation. Through these mechanisms, a given lymphocyte is able to modulate immune responses, destroy abnormal cells, or produce cytokines [29].

#### 6.1.1. The Mechanism of T-Lymphocyte Involvement in Hashimoto’s Thyroiditis

In Hashimoto’s thyroiditis, due to impaired immune surveillance, cells recognizing autoantigens are not eliminated in the selection process and migrate to peripheral tissues. It has been shown that autoreactive CD4+ T lymphocytes are activated and induce the influx of other cells into the thyroid. These include B and CD8+ T lymphocytes, which produce cytotoxic components such as perforin, granzymes, and proteoglycans [1].

T helper cells and regulatory T cells are important elements balancing the acquired immunity, mediating active immune responses or tolerance. Phenotypes of T helper lymphocytes involved in the pathogenesis of Hashimoto’s thyroiditis can be distinguished, such as Th1 and Th17 [7]. Upon stimulation by IL-12, IL-2, and IFN-γ, naïve CD4+ T lymphocytes differentiate into the Th1 subset, stimulating macrophages, B lymphocytes, and cytotoxic T lymphocytes (Tc) that damage the thyroid gland. They also produce factors that stimulate the immune system, such as IFN-γ, TNF-α, and IL-2. Interestingly, studies have shown that in patients with HT, MHC class II molecules can also be present on thyrocytes. The production of cytokines by Th1 lymphocytes, including IL-12 and IFN-y, induces the expression of MHC class II on thyroid cells and interferes with the differentiation process of T helper cells. Thyreocytes can act as APCs and present both autoantigens and foreign antigens to T lymphocytes, leading to their activation and enhancing the autoimmune process [27]. It has been shown that excessive stimulation of the immune response induced by Th1 is associated with the development of Hashimoto’s thyroiditis. These cells are responsible for inhibiting the growth and destruction of thyrocytes, inhibiting NIS, and reducing iodine binding to thyroglobulin. Recent studies have shown an association of Hashimoto’s thyroiditis with both Th1 and Th2 responses. Previously, it was claimed that HT was associated only with Th1 lymphocytes and Graves-Basedow disease with Th2. Th2 lymphocytes produce IL-4, IL-5, IL-6, IL-13, and stimulate B cells and plasma cells that produce antithyroid antibodies, which also contribute to inflammation. Although a Th1 lymphocyte-associated response predominates in HT, Th2 lymphocytes which promote a humoral immune response must also be considered in the pathogenesis of the disease [30].

#### 6.1.2. The Involvement of Th1 and Th17 in the Pathogenesis of Hashimoto’s Thyroiditis

The importance of Th1 lymphocytes is confirmed by studies in patients with autoimmune thyroid diseases, which have shown their predominance over Th2, and an intensified response of Th17 lymphocytes, leading to the exacerbation of inflammation in the body. In most of the analyses conducted, it was emphasized that the imbalance between Th1 and Th2 lymphocytes is the main element of the pathogenesis of the disease. Cytokines secreted by Th1 lymphocytes, with significant proinflammatory effects, have a crucial impact on the development of Hashimoto’s thyroiditis. The destruction of thyroid cells is mainly caused by the presence of IFN-γ, a cytokine associated with Th1, which plays a significant role in the immune response, the course of inflammation, and the increase in FAS expression on thyroid follicular cells in patients with Hashimoto’s thyroiditis [1]. It causes damage to the thyroid gland both directly and indirectly, and is also responsible for the increased activity in Th1 lymphocytes [31]. IL-23, which is produced by non-specific immune cells and influences the intensity of the Th1 response, may also be important in the pathogenesis of the disease [6]. Its role in Hashimoto’s thyroiditis disease is not clear, but the results of the research indicate an increase in the level of IL-23 in the blood of patients. The presence of this cytokine was found in 56% of patients with Hashimoto’s thyroiditis; therefore, further research is necessary to confirm its involvement [6].

Authors of recent studies have presented theories suggesting that the Th17 lymphocytes, regulatory T cells, and cytokines produced by these cells may be more important in the pathogenesis of HT. Th17 lymphocytes, which promote inflammation and produce cytokines, mainly IL-17, have been observed in recent years [32]. IL-17 is a key mediator of inflammation contributing to the development of the disease [3]. It has been shown to influence the severity of inflammation and the accumulation of lymphocytes, as well as the production of IL-1β, IL-6, and chemokines [1]. It has also been proven that IL-17 and the receptor associated with it, i.e., IL-17R, stimulate the signaling pathway dependent on nuclear factor κB (NF-κB), which plays an important role in the immune reaction by influencing cell differentiation and apoptosis. The importance of this interleukin in the pathogenesis of the disease was confirmed using clinical studies that showed an increased amount of IL-17 in patients suffering from Hashimoto’s thyroiditis. Importantly, the presence of this cytokine was noted not only in the blood of patients, but also in their thyroid tissue and in lymphocytic infiltration. The analysis of the thyrocytes in which it was found showed integrity disturbances and extensive damage, providing evidence of the relationship between IL-17 and the course of Hashimoto’s thyroiditis [3]. In autoimmune thyroid disease, a significant increase in the number of Th17 lymphocytes in the blood and their production of inflammatory mediators such as IL-17A, IL-17F, IL-21, and IL-22 have been observed [32]. As a result of the action of Th17, the activation of epithelial cells, macrophages, or fibroblasts occurs, leading to tissue damage, which is a characteristic manifestation of a disease resulting from auto-aggression [30]. It is also interesting to note the phenomenon whereby these lymphocytes are transformed into pathogenic cells called Th-1-like cells, which occurs as a result of chronic exposure to IL-23 [30]. It has been observed that the action of IL-23 and IL-17 initiates the production of further cytokines that play an important role in inflammation, such as TNF or IL-22 [7]. The influence of IL-23, 31, and 33 on the Th17/Treg lymphocyte ratio has also been demonstrated [33]. In a study conducted by Janyga et al., an increase in IL-23 and 31 was found in the group of people diagnosed with Hashimoto’s thyroiditis, confirming the involvement of IL-23 and 31 in the development of the disease [33]. In chronic inflammation, lymphocytes also show the ability to transform into Th1 under the influence of IL-12 [1]. Altered lymphocytes produce not only IFN-γ, but also a factor that stimulates generation of macrophages and granulocytes (GM-CSF); they also contribute to abnormalities at the molecular level [30]. Xue et al. demonstrated an association between macrophage migration inhibitory factor (MIF) levels and Th17 lymphocyte and IL-17A levels in patients with chronic lymphocytic thyroiditis [32]. MIF is a pro-inflammatory cytokine that affects both the innate and adaptive immune responses. Its influence on lymphocyte activation and pro-inflammatory cytokines has been documented. Recent analyses have shown that MIF is not only important in inflammation and infection, but also contributes to the development of Hashimoto’s thyroiditis by influencing the differentiation and development of Th17 lymphocytes [32]. In a study conducted by Vitales-Noyola et al., an imbalance was observed between Th17 cells that contribute to pathology and cells that belong to the same family but do not influence disease initiation. This finding may be useful in the diagnostic and therapeutic processes of diseases resulting from abnormal immune responses [34]. The importance of Th17 cells in the pathogenesis of the disease should be emphasized and considered in the development of new immunosuppressive agents. Initial studies with IL-17A-targeting drugs aim to halt or reduce the production of both Th17 lymphocytes and IL-17, potentially halting the development of autoimmune diseases [1].

#### 6.1.3. The Involvement of Th22 and Treg in the Pathogenesis of Hashimoto’s Thyroiditis

Another important subset of lymphocytes comprises Th22 cells, which, like Th17 cells, are capable of producing IL-22 [34]. When analyzing the importance of IL-22 in the pathogenesis of Hashimoto’s thyroiditis, it is worth paying attention to its relationship with the number of antibodies against thyroid peroxidase. Although IL-22 is more often associated with Graves’ disease, recent research suggests its possible role in Hashimoto’s thyroiditis. The studies conducted showed an increased amount of interleukin 17 and 22 in the blood of patients with chronic lymphocytic thyroiditis, meaning that a thorough analysis of the activity of these cytokines may provide new answers to questions regarding the pathogenesis of the disease [6,35]. In a study conducted by Vitales-Noyola et al., an increase in the number of Th22 lymphocytes was observed in patients with HT, along with their correlation with antithyroglobulin antibody levels. The authors suggested that these cells may play an important role in chronic inflammation [34]. The influence of IL-6 on the increase in differentiation into Th22 lymphocytes was also discovered; this should be taken into account in the pathogenesis of the disease [6].

Recent research also points to the involvement of regulatory T lymphocytes in autoimmune diseases. Reductions in their number and activity have been shown to be crucial in the development of autoimmunity. They are representatives of the CD3+ CD4+ helper T cell group, which has been divided into five subsets based on the expression of molecules. One recently discovered example is FoxP3+ CD69+ Treg, which provides tolerance and inhibits excessive immune responses by producing IL-10 and transforming growth factor-beta (TGF-beta) [3,30,36]. The results of many studies show the importance of FoxP3 expression, which is responsible for the development of Treg and its regulatory function. Abnormalities in this gene lead to Treg dysfunction and stimulation of T cells, initiating the disease process [3]. A study conducted by Chen et al. confirmed a significant decrease in both regulatory T lymphocyte levels and FoxP3 mRNA in the blood of newly diagnosed patients [3]. Tregs can also transform into Th1 and Th17 lymphocytes, which have been shown to directly correlate with increased autoimmunity and inflammation [30]. Many studies have shown their significant increase in patients compared to healthy individuals, suggesting a correlation with the severity of the disease [30,36]. Chen et al. also obtained results confirming this position, providing further evidence of the significant role of the Th17/Treg imbalance. The authors highlighted the importance of monitoring the levels of these cells, suggesting that they may be an effective diagnostic and therapeutic element in immune system disorders [3].

#### 6.1.4. The Involvement of Tfh in the Pathogenesis of Hashimoto’s Thyroiditis

Patients with chronic lymphocytic thyroiditis also show an increase in the number of Tfh belonging to the CD4+ T cell group, which play an important role in the immune response by producing IL-21 and stimulating B-lymphocyte activity [37]. They have been shown to be involved in the pathogenesis of many autoimmune diseases because the dysregulation of Tfh cell activity results in the increased production in antibodies against self-antigens. In a study conducted by Cai et al., an increased number in Tfh lymphocytes were found in the thyroid glands of patients with HT, which increased with the progression of the disease [37]. A correlation was also found between the number of these cells in the blood and thyroid and the level of antithyroid antibodies, especially anti-TG antibodies and free triiodothyronine. It has been suggested that, due to their importance in the development of autoimmune thyroid diseases, Tfh lymphocytes may serve as markers indicating the presence of these diseases and allowing for the assessment of their progression [37].

#### 6.1.5. The Involvement of Tc in the Pathogenesis of Hashimoto’s Thyroiditis

Cytotoxic T lymphocytes are also considered to be important elements in the pathogenesis of Hashimoto’s thyroiditis. Their role is to protect the body from threats such as viruses or tumors. With the presence of the TCR receptor and the expression of the CD8+ protein, they are able to kill cells with abnormal antigens presented in MHC class I molecules [38]. During the progression of autoimmune diseases, production of CD8+ T lymphocytes is increased due to the loss of control over autoreactive CD4+ T lymphocytes. Recent studies have shown that the ratio of CD4+ to CD8+ cells also correlates with disease progression. These cells are able to produce perforin, granzymes such as granzyme B, and proteoglycans in the thyroid gland, leading to thyrocytes’ cytotoxic death, gland destruction, and chronic inflammation [27,29]. Analysis has confirmed increased numbers of cytotoxic T cells in the blood of patients, suggesting their significant role in disease pathogenesis. Although many clinical studies confirm the involvement of T lymphocytes in pathogenesis, and their general mechanisms of action are well understood, it is still uncertain which subpopulation is a key to pathogenesis of a disease. Further analysis may shed light on this issue.

### 6.2. B Lymphocytes

Effector cells of the humoral response also play a critical role in the pathogenesis of Hashimoto’s thyroiditis [27]. Each B lymphocyte is produced in the bone marrow, and undergoes a partial selection process before maturing [39]. A loss of tolerance leads to the activation of B lymphocytes that recognize autoantigens, initiating the autoimmune process [40]. In patients with HT, autoreactive B lymphocytes, as plasma cells, are capable of producing antithyroid antibodies and cytokines, leading to increased inflammation. However, this is not their only role in disease pathogenesis. They can also present antigens and stimulate naive CD4+ T lymphocytes to recognize thyroid antigens. When discussing the importance of B lymphocytes, attention should also be paid to the influence of CD4+ T lymphocytes on their activity. It has been shown that, after binding to TG epitopes, they influence the process whereby B lymphocytes are differentiated into plasma cells [41]. Observations confirm the presence of B lymphocytes in the thyroid gland and their involvement in the development of the disease. Patients with autoimmune thyroid diseases, including Hashimoto’s thyroiditis, have also been observed to have reduced numbers in anergic TG-specific B cells in the blood [41]. Similar results were obtained in a study conducted by Smith et al., where a decreased total number of anergic B cells and those capable of reacting with TG and TPO, as well as higher levels of antibodies against self-antigens, were found in the blood of newly diagnosed patients compared to those with a longer history of the disease or those without the disease [40].

**Figure 2:**
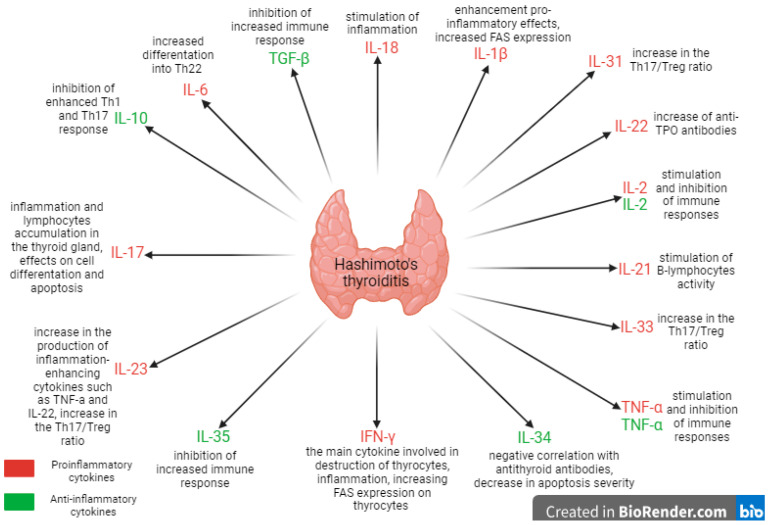
The involvement of cytokines in Hashimoto’s thyroiditis (created using BioRender.com, https://www.biorender.com/, accessed on 20 April 2024).

The development of autoimmune diseases also highlights the special role of regulatory B cells (Bregs), mainly B10, which have been shown to inhibit the enhanced Th1 and Th17 responses and induce the disease remission through IL-10 production (Figure 2). Deficiency of these lymphocytes has been associated with the progression of autoimmune diseases [39]. The ability of regulatory B lymphocytes to produce the cytokine IL-35, which inhibits excessive immune responses, has also been demonstrated [39]. An interesting discovery relates to the influence of lipopolysaccharide (LPS) on IL-10-producing Bregs. It has been shown that the combined effect of LPS and antibodies against CD40 is significant in the stimulation of B lymphocytes and results in the increased activity of Bregs-releasing IL-35 [39]. Studies have also shown the influence of regulatory B lymphocytes in reducing cell differentiation, increasing FOXP3 and CTLA-4 expression in regulatory T cells, and inhibiting cytokine production such as that of TNF-α and IFN-γ by CD4+ T lymphocytes [1]. Although the impact of B lymphocytes on the development of Hashimoto’s thyroiditis is becoming more thoroughly understood, there are still a lack of proposals on how knowledge of their activity can be implemented in the therapeutic process. Blocking or inhibiting the activity of B lymphocyte-recognizing autoantigens may be beneficial in eliminating disease symptoms, so it is worth considering these mechanisms when developing new therapeutic solutions [41]. In the study conducted by Ralchev et al., the authors suggest that the activity of B lymphocytes could be effectively reduced by using complement receptors [41]. Regulatory B cells may prove to be beneficial in the strategies in patients with autoimmune diseases; therefore, further research into the mechanisms affecting their production and activity is essential.

### 6.3. Natural Killer Cells

Recent studies have repeatedly demonstrated the influence of NK cells on the regulation of the immune system and autoimmune diseases. NK cells are generated in the bone marrow and belong to the elements of non-specific immunity. They monitor the tissues and check whether all cells properly present antigens in MHC class I molecules [28,38]. They have a direct cytotoxic effect on abnormal cells, such as cancerous or infected cells, as well as autoreactive cells. Although NK cells are considered to be among the first responders in the defense mechanism when autoimmunity arises, their action is not always effective. It has been shown that an increased attack on abnormal cells that have appeared in the body can result in damage to NK cells and the production of autoantigens. The consequence of such a phenomenon is the stimulation of lymphocytes recognizing self-antigens and the initiation of the autoimmune process. NK cells play an important role across different stages of Hashimoto’s thyroiditis. During auto-aggression, when immune system dysfunction is observed, there is increased activation of the antibody-dependent immune response, which activates NK cells [42]. They participate in antibody-dependent cell-mediated cytotoxicity (ADCC) and have the ability to release granules containing perforin and granzymes, leading to the destruction of thyrocytes [28,42]. NK cells also influence dendritic cell activity and antigen presentation and attenuate the effects of B and T lymphocytes. The production of pro-inflammatory mediators such as IFN-γ stimulates macrophages and promotes the dominance of the Th1 response, which is prevalent in HT. A study conducted by Martin et al. demonstrated the existence of several distinct NK cell subpopulations with immunological activity that, when measured in the blood of patients along with antibody and thyrotropin levels, may be useful in the diagnosis of Hashimoto’s thyroiditis [42].

## 7. Importance of Apoptosis in the Pathogenesis of Hashimoto’s Thyroiditis

Apoptosis is a crucial physiological mechanism that effectively regulates the immune response. It plays a vital role in eliminating non-functional or autoantigen-recognizing cells [43]. In the context of Hashimoto’s thyroiditis, the disease pathogenesis is characterized by the infiltration of immune system cells into the thyroid gland, leading to the death of thyrocytes. The regulation of apoptosis involves various factors, such as TSH, anti-TPO antibodies, and inflammatory mediators such as IFN-γ and IL-2 [43]. The Fas apoptosis-inducing receptor (FAS), which possesses a death domain, plays a significant role in this process through death receptors. Hashimoto’s thyroiditis has been found to increase the expression of FAS molecules, leading to the apoptosis of thyrocytes. This is a significant finding, as it highlights the externally initiated mechanism that triggers programmed cell death [1]. In the most recent analyses, the authors provide evidence for the importance of IL-34 in the course of apoptosis in Hashimoto’s thyroiditis [31]. Wang et al. observed the reduced expression in this interleukin in the thyroid tissue, as well as a decrease in its level in the blood of patients. The negative correlation of IL-34 with antibodies against thyroid peroxidase and thyroglobulin was also demonstrated, as well as an effect on reducing the severity of the process of apoptosis [31].

### The Regulation of Apoptosis

**Figure 3:**
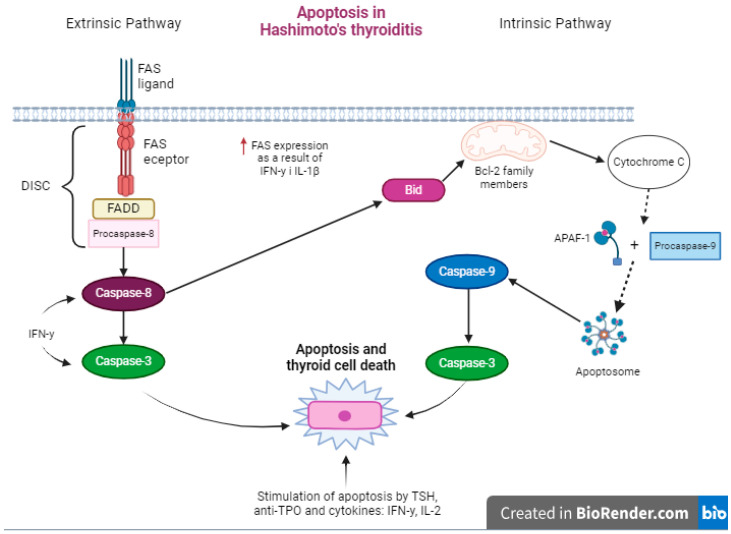
The course of apoptosis in Hashimoto’s thyroiditis (created using BioRender.com, https://www.biorender.com/, accessed on 20 April 2024). ↑—increase in synthesis.

The FAS ligand (FasL) binds to the receptor and interacts with proteins, including the FAS-associated death domain-containing protein (FADD), procaspase-8 recruitment, and the formation of the death-inducing signaling complex (DISC) [35]. The involvement of FAS and FAS ligands in initiating apoptosis is widely acknowledged by researchers. Cytokines such as IFN-γ and IL-1β play a crucial role in increasing receptor expression (Figure 3), which exacerbates the damage to thyroid follicular cells and oxidative stress [1,7]. Moreover, proteins from the Bcl-2 family can activate intrinsic apoptotic pathways. The activation of caspases, such as caspase-8, can result in the release of cytochrome C and the formation of an apoptosome composed of cytochrome C, apoptotic protease-activating factor 1 (APAF-1), and procaspase-9. This triggers the activation of caspase-9, which enhances the action of caspase-3, one of the most important effector caspases in programmed cell death [31]. Additionally, the intrinsic pathway leading to apoptosis through DNA damage, metabolic disturbances, and abnormal cell cycle progression is well known. These mechanisms ultimately lead to cell death [35]. Research has demonstrated a clear association between FAS gene polymorphisms and the development of Hashimoto’s thyroiditis as a result of mutations that lead to the accumulation of T lymphocytes [1]. The role of intra-thyroidal T lymphocytes in apoptosis is significant in patients with chronic lymphocytic thyroiditis. The influence of these lymphocytes on regulating the expression of proapoptotic molecules via cytokines has been well established. These cells express Fas/FasL genes [43]. Increased FAS expression and elevated levels of procaspases have been linked to cytokines produced by macrophages and Th1 lymphocytes [35]. Patients also exhibit the increased expression of proteins such as Bid and Bak, which stimulate apoptosis. Disease progression is influenced by both proapoptotic and antiapoptotic molecules [43]. The Bcl-2 protein group significantly inhibits apoptosis. In patients with HT, Bcl-2 levels are lower than in healthy individuals [35]. The disease leads to the excessive activation of pathways that cause thyrocyte death and disrupt apoptosis regulatory mechanisms, which could prevent cell destruction. It is worth noting that thyroid hormones have also been observed to influence reduced Bcl-2 expression [43]. The level of antibodies against thyroid peroxidase significantly affects the progression of apoptosis. This association is due to T lymphocyte resistance to FAS-induced programmed cell death. It is important to note that this mechanism occurs only in patients who are not undergoing thyroid hormone therapy [35]. IFN-γ activates gene expression that enhances apoptosis and caspase 3 and 8, resulting in damage to the thyroid gland and the impairment of its function [1].

## 8. Inflammation in Hashimoto’s Thyroiditis

### 8.1. Antibodies

Over the course of Hashimoto’s thyroiditis, in addition to the cellular response, the humoral response plays a significant role. This involves the production of antibodies against thyroid peroxidase and thyroglobulin, which are considered to be characteristic manifestations of the disease and occur in the majority of patients [7]. The particular involvement of anti-TPO in the pathogenesis of the disease is emphasized, as confirmed by studies showing a correlation between these antibodies and the number of lymphocytes infiltrating the thyroid gland. Anti-TPO antibodies lead to gland damage through the following two types of cytotoxicity: antibody-dependent cell-mediated cytotoxicity, and complement-dependent cytotoxicity [7,10]. Damaged thyrocytes start releasing cytokines such as IL-6, IL-1β, and IL-8, resulting in increased inflammation and the accumulation of lymphocytes within the thyroid gland. Kristensen’s work also presented the involvement of antithyroid peroxidase antibodies in cytokine production by phagocytes and T lymphocytes, which promote inflammation [1]. The significance of anti-TG in the pathogenesis of Hashimoto’s thyroiditis is not fully understood. Studies in animals have shown that they appear in the bloodstream faster than anti-TPO antibodies, indicating an earlier change in B and T lymphocyte tolerance towards thyroglobulin [10]. Kristensen described another role of the thyroid autoantigen TG, which is capable of initiating IL-10 and IL-6 production by B lymphocytes, as well as IL-10 by T lymphocytes [1]. In the latest research on the pathogenesis of Hashimoto’s thyroiditis, the influence of MIF on antibodies occurring over the course of the disease has also been demonstrated. Xue et al. first proved that mRNA expression and the described factor showed a positive correlation with antithyroid antibodies and TSH levels in the blood, confirming the influence of MIF on the development of autoimmune thyroid diseases [32]. It is also worth emphasizing the importance of exosomes, which were discovered to influence the initiation of autoimmune diseases. They are responsible for transporting the MHC-IITPO/Tg complex to dendritic cells. This leads to antigen acceptance and disturbance in the differentiation of CD4+ cells, resulting in disease initiation [7]. The thyroid gland can be attacked not only by lymphocytes, but also by immunoglobulin G (IgG) that is IgG4 positive. It was observed that there were significantly higher numbers of these cells in patients with HT, a phenomenon associated with a more rapid progression of thyroid dysfunction [7]. Other elements that can be distinguished in the pathogenesis of chronic lymphocytic thyroiditis are antibodies against the sodium-iodide symporter, which is responsible for limited iodine transport out of the cell [7]. Although the involvement of antibodies in the development of Hashimoto’s thyroiditis is certain, there is still a lack of evidence as to whether they are responsible for initiating an abnormal immune response or appear secondarily. The level of antibodies against thyroid peroxidase and thyroglobulin can be used to assess the severity of the condition [32].

### 8.2. Highly Inflammatory Cell Death—Pyroptosis

In terms of pathogenetic mechanisms, many cytokines have been identified that affect thyroid cells in the periphery to enhance pro-inflammatory effects. It appears that the expression of inflammasome components and a number of defects in regulatory T cells underlie the loss of self-tolerance to thyroid autoantigens [44]. Inflammasomes are protein complexes responsible for initiating intracellular inflammatory processes. Inflammasomes not only play a regulatory role, but also influence the course of autoimmune diseases and carcinogenesis. They are also involved in the innate immune response as functional receptors. Activation of inflammasomes in autoimmune thyroiditis occurs through the microRNA-612/BRD4 axis, and LINC01061 can promote the inflammatory response of thyroid follicular epithelial cells [45].

The role of the inflammasome is to activate caspase 1 and initiate the process of pyroptosis. Meanwhile, the NLR family CARD domain-containing 4 (NLRC4) belongs to the NLR family and is mainly activated by Gram-negative bacteria [46]. Pyroptosis gives the impression that the immune system has finally outsmarted pathogenic microorganisms. Recently, a significant association of NLRC4 rs385076 with Hashimoto’s thyroiditis was identified for the first time [46]. Inflammasomes, which mediate the maturation of interleukin-1β (IL-1β) and interleukin-18 (IL-18), are of fundamental importance in autoimmune thyroid diseases (AIT) [47]. The results indicate a significant effect of IL-1β rs1143634 and COX-2 (rs2745557) SNPs in the development of HT [48].

The expression of NLRP1, NLRP3, NLRC4, and AIM2 was mainly localized in thyroid follicular cells adjacent to areas of lymphocytic infiltration. Thyroid NLRP1 and ASC mRNA levels correlated with TPOAb and TgAb levels. TNF-α and IFN-γ stimulated the expression of many inflammasome components in thyroid cells. IFN-γ was found to enhance cell pyroptosis. The identified inflammasomes NLRP3, NLRP1, NLRC4, AIM2, and their downstream cytokines may serve as potential therapeutic targets and biomarkers of AIT [47].

**Figure 4:**
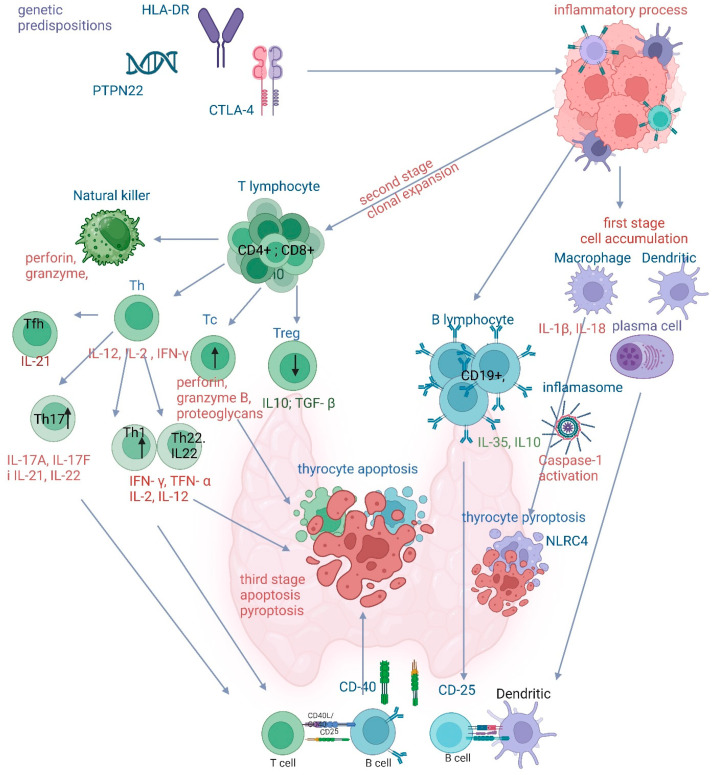
The role of the immune system in the progression of Hashimoto’s thyroiditis. CTLA-4—antibodies for cytotoxic T-cell antigen 4; HLA-DR—human leukocyte antigens class II; PTPN22—protein tyrosine phosphatase non-receptor type 22; NK—natural killer; Tfh—T follicular helper cells; Th—T helper cells; Tc—cytotoxic T lymphocytes; Treg—regulatory T cells; ↑ or ↓—decrease or increase in synthesis (created using BioRender.com, https://app.biorender.com/, accessed on 4 April 2024).

The current state of knowledge regarding the involvement of the immune system in the pathogenesis of Hashimoto’s thyroiditis is summarized in Figure 4.

## 9. Research Methods and Techniques

**Figure 5:**
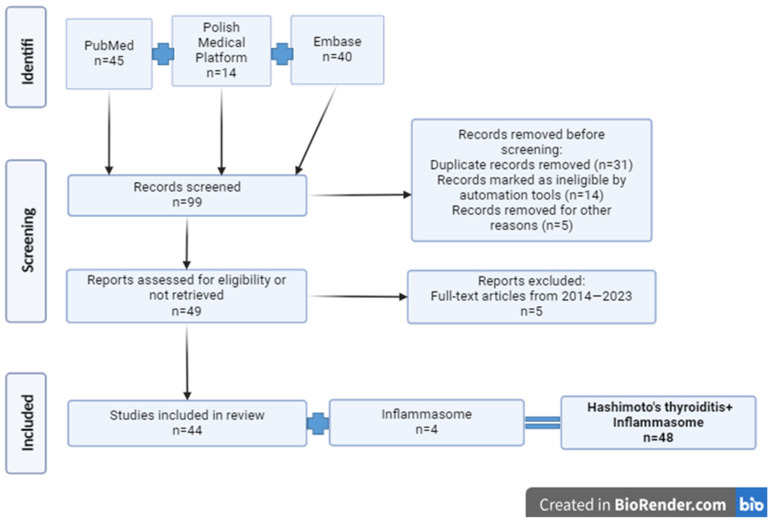
Selection of articles (created using BioRender.com, https://www.biorender.com/, accessed on 11 June 2024).

This article uses the literature review method. To find scientific articles, three databases of scientific publications were used: PubMed, the Polish Medical Platform, and Embase. Scientific articles were identified using the following phrases in various combinations: “Thyroid gland”, “Hashimoto’s thyroiditis”, “Immune system”, “Autoimmunity”, “Autoimmune thyroiditis”, “Lymphocytes”, “Antibodies”, “T-cells”, and “B-cells”. The work uses Polish and foreign research from 2014–2023, for a total of 48 items. The selection of articles is presented in Figure 5.

## References

[1] Kristensen B. (2016). Regulatory B and T cell responses in patients with autoimmune thyroid disease and healthy controls. Dan. Med. J..

[2] Ihnatowicz P., Drywień M., Wątor P., Wojsiat J. (2020). The importance of nutritional factors and dietary management of Hashimoto’s thyroiditis. Ann. Agric. Environ. Med..

[3] Chen A., Huang L., Zhang L. (2023). Helper T Cell 17 and Regulatory T Cell Levels in Peripheral Blood of Newly Diagnosed Patients with Autoimmune Thyroid Disease: A Meta-Analysis. Horm. Metab. Res..

[4] Salazar-Viedma M., Vergaño-Salazar J.G., Pastenes L., D’Afonseca V. (2021). Simulation Model for Hashimoto Autoimmune Thyroiditis Disease. Endocrinology.

[5] Klubo-Gwiezdzinska J., Wartofsky L. (2022). Hashimoto thyroiditis: An evidence-based guide to etiology, diagnosis and treatment. Pol. Arch. Intern. Med..

[6] Ajjan R.A., Weetman A.P. (2015). The Pathogenesis of Hashimoto’s Thyroiditis: Further Developments in our Understanding. Horm. Metab. Res..

[7] Jin B., Wang S., Fan Z. (2022). Pathogenesis Markers of Hashimoto’s Disease—A Mini Review. Front. Biosci..

[8] Mikulska A.A., Karaźniewicz-Łada M., Filipowicz D., Ruchała M., Główka F.K. (2022). Metabolic Characteristics of Hashimoto’s Thyroiditis Patients and the Role of Microelements and Diet in the Disease Management—An Overview. Int. J. Mol. Sci..

[9] Gąbka I., Dalmata W., Gendek K., Dąbrowski J., Kozłowska A., Korzeniowska A., Załęska N., Ziółkiewicz A. (2023). Hashimoto’s disease—The role of factors and diet in the course of the disease. J. Educ. Health Sport.

[10] Vargas-Uricoechea H. (2023). Molecular Mechanisms in Autoimmune Thyroid Disease. Cells.

[11] Batóg G., Dołoto A., Bąk E., Piątkowska-Chmiel I., Krawiec P., Pac-Kożuchowska E., Herbet M. (2023). The interplay of oxidative stress and immune dysfunction in Hashimoto’s thyroiditis and polycystic ovary syndrome: A comprehensive review. Front. Immunol..

[12] Kosiak W., Piskunowicz M., Świętoń D., Batko T., Kaszubowski M. (2015). An additional ultrasonographic sign of Hashimoto’s lymphocytic thyroiditis in children. J. Ultrason..

[13] Thomas T., Sreedharan S., Khadilkar U.N., Deviprasad D., Kamath M.P., Bhojwani K.M., Alva A. (2014). Clinical, biochemical & cytomorphologic study on Hashimoto’s thyroiditis. Indian J. Med. Res..

[14] Yuan J., Qi S., Zhang X., Lai H., Li X., Xiaoheng C., Li Z., Yao S., Ding Z. (2023). Local symptoms of Hashimoto’s thyroiditis: A systematic review. Front. Endocrinol..

[15] Groenewegen K.L., Mooij C.F., van Trotsenburg A.S.P. (2021). Persisting symptoms in patients with Hashimoto’s disease despite normal thyroid hormone levels: Does thyroid autoimmunity play a role? A systematic review. J. Transl. Autoimmun..

[16] Staruszkiewicz M., Pituch-Noworolska A., Skoczen S. (2023). SARS-CoV-2 and thyroid diseases. J. Transl. Autoimmun..

[17] Szaryńska M. (2020). Comment on: Molecular Functions of Thyroid Hormone Signaling in Regulation of Cancer Progression and Anti-Apoptosis. Int. J. Mol. Sci..

[18] Wawrzyniak S., Rakoca M., Kułakowska A., Bartosik-Psujek H., Koziarska D., Kapica-Topczewska K., Kubicka-Bączyk K., Adamczyk-Sowa M. (2023). Multiple sclerosis and autoimmune diseases—A case control study. Neurol. Neurochir. Pol..

[19] Morawiec-Szymonik E., Foltyn W., Marek B., Kos-Kudła B., Kajdaniuk D. (2019). Pernicious anaemia and endocrine glands antibodies. Endokrynol. Pol..

[20] Betterle C., Furmaniak J., Sabbadin C., Scaroni C., Presotto F. (2023). Type 3 autoimmune polyglandular syndrome (APS-3) or type 3 multiple autoimmune syndrome (MAS-3): An expanding galaxy. J. Endocrinol. Investig..

[21] Jankowska K., Dudek P., Stasiek M., Suchta K. (2023). Autoimmune polyendocrine syndromes associated with autoimmune rheumatic diseases. Reumatologia.

[22] Ashok T., Patni N., Fatima M., Lamis A., Siddiqui S.W. (2022). Celiac Disease and Autoimmune Thyroid Disease: The Two Peas in a Pod. Cureus.

[23] Kayar Y., Dertli R. (2019). Association of autoimmune diseases with celiac disease and its risk factors. Pak. J. Med. Sci..

[24] Szeliga A., Calik-Ksepka A., Maciejewska-Jeske M., Grymowicz M., Smolarczyk K., Kostrzak A., Smolarczyk R., Rudnicka E., Meczekalski B. (2021). Autoimmune Diseases in Patients with Premature Ovarian Insufficiency-Our Current State of Knowledge. Int. J. Mol. Sci..

[25] Szczuko M., Syrenicz A., Szymkowiak K., Przybylska A., Szczuko U., Pobłocki J., Kulpa D. (2022). Doubtful Justification of the Gluten-Free Diet in the Course of Hashimoto’s Disease. Nutrients.

[26] Mikosch P., Aistleitner A., Oehrlein M., Trifina-Mikosch E. (2023). Hashimoto’s thyroiditis and coexisting disorders in correlation with HLA status-an overview. Wien. Med. Wochenschr..

[27] Luty J., Ruckemann-Dziurdzińska K., Witkowski J.M., Bryl E. (2019). Immunological aspects of autoimmune thyroid disease—Complex interplay between cells and cytokines. Cytokine.

[28] Popko K., Górska E. (2015). The role of natural killer cells in pathogenesis of autoimmune diseases. Cent. Eur. J. Immunol..

[29] Sun L., Su Y., Jiao A., Wang X., Zhang B. (2023). T cells in health and disease. Signal Transduct. Target. Ther..

[30] Rydzewska M., Jaromin M., Pasierowska I.E., Stożek K., Bossowski A. (2018). Role of the T and B lymphocytes in pathogenesis of autoimmune thyroid diseases. Thyroid Res..

[31] Wang S., Liu Y., Zhao N., Cui X., Huang M., Li Y., Shan Z., Teng W. (2018). IL-34 Expression Is Reduced in Hashimoto’s Thyroiditis and Associated With Thyrocyte Apoptosis. Front. Endocrinol..

[32] Xue H., Yang Y., Zhang Y., Song S., Zhang L., Ma L., Yang T., Liu H. (2015). Macrophage migration inhibitory factor interacting with Th17 cells may be involved in the pathogenesis of autoimmune damage in Hashimoto’s thyroiditis. Mediat. Inflamm..

[33] Janyga S., Kajdaniuk D., Czuba Z., Ogrodowczyk-Bobik M., Urbanek A., Kos-Kudła B., Marek B. (2023). Interleukin (IL)-23, IL-31, and IL-33 Play a Role in the Course of Autoimmune Endocrine Diseases. Endocr. Metab. Immune Disord. Drug Targets.

[34] Vitales-Noyola M., Ramos-Levi A.M., Martínez-Hernández R., Serrano-Somavilla A., Sampedro-Nuñez M., González-Amaro R., Marazuela M. (2017). Pathogenic Th17 and Th22 cells are increased in patients with autoimmune thyroid disorders. Endocrine.

[35] Pyzik A., Grywalska E., Matyjaszek-Matuszek B., Roliński J. (2015). Immune disorders in Hashimoto’s thyroiditis: What do we know so far?. J. Immunol. Res..

[36] Mazzieri A., Montanucci P., Basta G., Calafiore R. (2022). The role behind the scenes of Tregs and Th17s in Hashimoto’s thyroiditis: Toward a pivotal role of FOXP3 and BACH2. Front. Immunol..

[37] Cai Y., Wang Z., Liu X., Wei L., Li S., Zheng X., Yang T., Xu X. (2022). The Frequency of Intrathyroidal Follicular Helper T Cells Varies with the Progression of Graves’ Disease and Hashimoto’s Thyroiditis. J. Immunol. Res..

[38] Nicholson L.B. (2016). The immune system. Essays Biochem..

[39] Egwuagu C.E., Yu C.R. (2015). Interleukin 35-Producing B Cells (i35-Breg): A New Mediator of Regulatory B-Cell Functions in CNS Autoimmune Diseases. Crit. Rev. Immunol..

[40] Smith M.J., Rihanek M., Coleman B.M., Gottlieb P.A., Sarapura V.D., Cambier J.C. (2018). Activation of thyroid antigen-reactive B cells in recent onset autoimmune thyroid disease patients. J. Autoimmun..

[41] Ralchev N.R., Markovski A.M., Yankova I.A., Manoylov I.K., Doytchinova I.A., Mihaylova N.M., Shinkov A.D., Tchorbanov A.I. (2022). Selective Silencing of Disease-Associated B Lymphocytes from Hashimoto’s Thyroiditis Patients by Chimeric Protein Molecules. Int. J. Mol. Sci..

[42] Martin T.C., Ilieva K.M., Visconti A., Beaumont M., Kiddle S.J., Dobson R.J.B., Mangino M., Lim E.M., Pezer M., Steves C.J. (2020). Dysregulated Antibody, Natural Killer Cell and Immune Mediator Profiles in Autoimmune Thyroid Diseases. Cells.

[43] Krawczyk A., Miśkiewicz J., Strzelec K., Wcisło-Dziadecka D., Strzalka-Mrozik B. (2020). Apoptosis in Autoimmunological Diseases, with Particular Consideration of Molecular Aspects of Psoriasis. Med. Sci. Monit..

[44] Weetman A.P. (2021). An update on the pathogenesis of Hashimoto’s thyroiditis. J. Endocrinol. Investig..

[45] Zhao Y., Xu L., Wang Q., Li C., Zhang T., Xing S., Yu X. (2022). LINC01061 triggers inflammation and inflammasome activation in autoimmune thyroiditis via microRNA-612/BRD4 axis. Int. Immunopharmacol..

[46] Liu X., Bai X., Zhao J., Gao C., Du P., Zhang J.A., Li S. (2020). Associations between NLRC4 Gene Polymorphisms and Autoimmune Thyroid Disease. Biomed. Res. Int..

[47] Guo Q., Wu Y., Hou Y., Liu Y., Liu T., Zhang H., Fan C., Guan H., Li Y., Shan Z. (2018). Cytokine Secretion and Pyroptosis of Thyroid Follicular Cells Mediated by Enhanced NLRP3, NLRP1, NLRC4, and AIM2 Inflammasomes Are Associated With Autoimmune Thyroiditis. Front. Immunol..

[48] Heidari Z., Salimi S., Rokni M., Rezaei M., Khalafi N., Shahroudi M.J., Dehghan A., Saravani M. (2021). Association of IL-1β, NLRP3, and COX-2 Gene Polymorphisms with Autoimmune Thyroid Disease Risk and Clinical Features in the Iranian Population. Biomed. Res. Int..

